# Thalamic neuromodulation and its implications for executive networks

**DOI:** 10.3389/fncir.2014.00069

**Published:** 2014-06-24

**Authors:** Carmen Varela

**Affiliations:** Picower Institute for Learning and Memory, Massachusetts Institute of TechnologyCambridge, MA, USA

**Keywords:** modulators, acetylcholine, serotonin, dopamine, noradrenaline, histamine, midline, intralaminar

## Abstract

The thalamus is a key structure that controls the routing of information in the brain. Understanding modulation at the thalamic level is critical to understanding the flow of information to brain regions involved in cognitive functions, such as the neocortex, the hippocampus, and the basal ganglia. Modulators contribute the majority of synapses that thalamic cells receive, and the highest fraction of modulator synapses is found in thalamic nuclei interconnected with higher order cortical regions. In addition, disruption of modulators often translates into disabling disorders of executive behavior. However, modulation in thalamic nuclei such as the midline and intralaminar groups, which are interconnected with forebrain executive regions, has received little attention compared to sensory nuclei. Thalamic modulators are heterogeneous in regards to their origin, the neurotransmitter they use, and the effect on thalamic cells. Modulators also share some features, such as having small terminal boutons and activating metabotropic receptors on the cells they contact. I will review anatomical and physiological data on thalamic modulators with these goals: first, determine to what extent the evidence supports similar modulator functions across thalamic nuclei; and second, discuss the current evidence on modulation in the midline and intralaminar nuclei in relation to their role in executive function.

## INTRODUCTION AND KEY TERMS

### THALAMIC AFFERENTS: DRIVERS AND MODULATORS

All the forebrain structures that contribute to cognitive functions receive input from the thalamus, which is a critical point for the routing of information and gateway control. Thalamic cells receive two general types of afferents, drivers and modulators. Thalamic drivers are afferents that target proximal dendrites with relatively large synaptic boutons, reliably evoke spikes in thalamic cells, and whose function is thought to be the faithful transmission of the spike message relayed by thalamic cells to postsynaptic structures. In contrast, modulators are those afferents that target primarily distal dendrites and influence spike transmission by adjusting the cellular and synaptic mechanisms underlying spike generation; by doing so, they are thought to fine-tune the message relayed by thalamic cells and control its probability of transmission (reviewed in Sherman and Guillery, 1998; Guillery and Sherman, 2002). It should be noted that this distinction between drivers and modulators is largely based on evidence from the sensory thalamus, which has critical relay functions. Outside of the sensory thalamus, the evidence (still scarce and mostly anatomical) suggests that the anatomical features that distinguish drivers and modulators are present in all thalamic nuclei, although the functional correlates regarding spike generation and transmission still need to be characterized for many thalamic regions. For example, nuclei outside the primary sensory thalamus receive afferents with driver morphology from multiple sources (Baldauf et al., 2005; Masterson et al., 2009). These drivers converging onto individual cells may contribute to spike generation like the drivers in sensory thalamus, but each of them could also contribute to subthreshold modulation that is integrated across all drivers to generate an output, something that will need to be tested. Similarly, some modulators outside of the primary sensory thalamus share features of drivers (such as the large cholinergic afferents in some higher order nuclei, reviewed below). Therefore, the definition of drivers and modulators that is used here is an operational definition that may need refinement as we learn more about the thalamus. 

In every thalamic nucleus studied to date, modulator synapses are found to constitute the vast majority of inputs to a given relay cell. The innervation by modulators is particularly dense in the midline and intralaminar groups of thalamic nuclei, both interconnected with executive areas such as the medial prefrontal cortex (mPFC) and basal ganglia. mPFC and the basal ganglia have been extensively studied, including the effect of modulators on these regions. Surprisingly, the midline and intralaminar nuclei are largely unexplored territory compared to neocortex, basal ganglia, or the sensory thalamic nuclei. Even some basic questions, such as the cell response properties or the modulator effects on these thalamic nuclei, remain unanswered. This review will first discuss anatomical and physiological results on modulators across the thalamus. In the second part, it will review recent evidence that highlights the importance of midline and intralaminar nuclei in executive functions, and the role of modulators in these nuclei. The objective is to point out important gaps in knowledge and untested hypotheses regarding the function of modulators in the thalamus. Recent technological developments (optogenetics, pharmacogenetics, clearing techniques such as “clarity”) provide powerful tools to address many open questions that must be answered in order to elucidate the role of thalamic modulation in executive networks.

**FIGURE 1:**
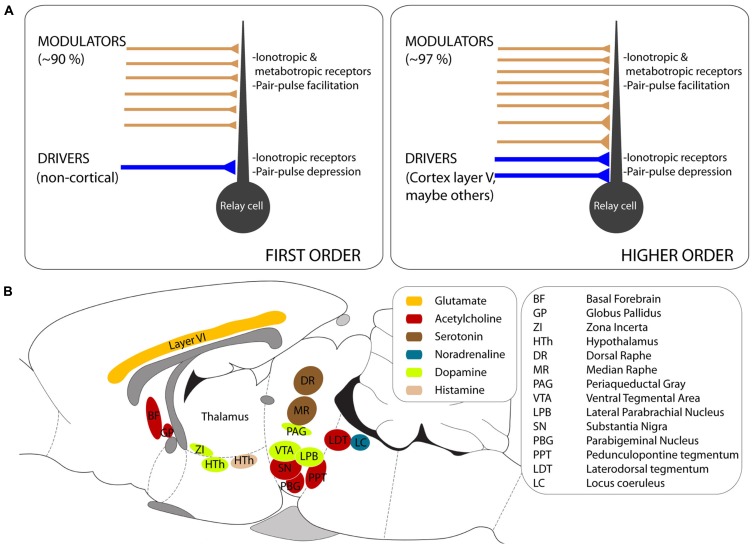
**General properties of thalamic afferents. (A)** Schematic representation of the features that distinguish drivers and modulators in first order (left) and higher order (right) nuclei. Note the higher fraction of modulators in higher order nuclei, where some modulators have large synapses contacting proximal dendrites. In both first and higher order nuclei, modulators activate ionotropic and metabotropic receptors and, in those in which it has been tested, the postsynaptic responses facilitate under repetitive stimulation. **(B)** Approximate location of the brain regions that provide modulator afferents to the thalamus, color-coded for the neurotransmitter they use. The outline of a rodent brain is used for convenience, although the diagram combines results from different species (see text for details).

Modulators constitute a heterogeneous group of afferents that nevertheless share some anatomical and physiological properties across the thalamus (reviewed in Sherman and Guillery, 1998; **Figure 1A**). Modulators originate in a variety of brain regions and use various neurotransmitters (summarized schematically in **Figure 1B**). Examples of thalamic modulators include cholinergic, serotonergic, dopaminergic, and noradrenergic afferents from the brainstem, histaminergic afferents from the hypothalamus, and glutamatergic afferents from layer VI of neocortex.

The first part of this review will discuss the anatomy and physiology of six chemically defined modulators across the thalamus. GABAergic inputs to thalamic nuclei, which originate primarily from diencephalic sources, will not be considered in this review. Furthermore, many neuroactive peptides (including orexins) co-localize with neurotransmitter systems in the thalamus (reviewed in Jones, 2007), and can have wider effects than neurotransmitters, for example, on gene expression, synaptogenesis, local blood flow, etc. Because of their broad spectrum of actions they fall far from the scope of this review. Similarly, other unconventional neurotransmitters like endocannabinoids, purines, and nitric oxide are present in the terminals of some thalamic afferents (reviewed in Jones, 2007), but their effects will not be examined here.

### THALAMIC NUCLEI: FIRST AND HIGHER ORDER

**FIGURE 2:**
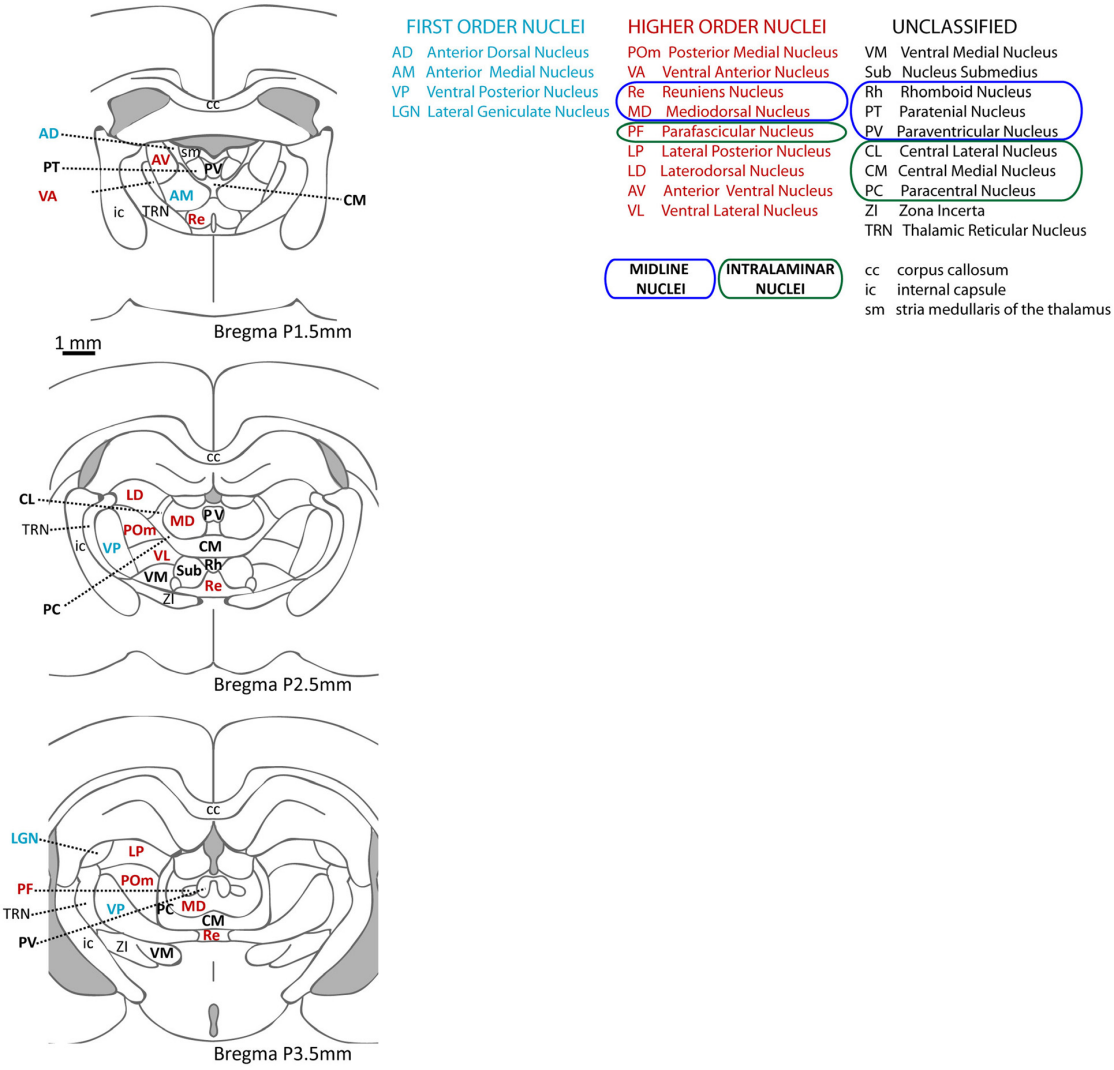
**Thalamic nuclei in the rat**. Schematic representation of the nuclei in the rat thalamus at three different anteroposterior levels (based on Paxinos and Watson, 2004) and their abbreviated names. First order nuclei are labeled in blue, higher order in red, and nuclei that have not been classified as first or higher order are in black. Note that the diagrams do not include the medial geniculate nucleus (more posterior), which includes a first order region (the ventral portion) as well as higher order regions (dorsal portion).

Guillery (1995) distinguished two groups of thalamic nuclei: “First order” are those nuclei that receive drivers from ascending afferent pathways, and transmit information that arrives at the thalamus for the first time. Nuclei in the other group were named “higher order,” and are those that relay information that has gone through the thalamus at least once (through a first order nucleus). The main feature that distinguishes higher from first order nuclei is that at least some of their driver input originates in layer V of neocortex; for this reason, they are thought to participate in cortico-cortical communication (Theyel et al., 2010). The first order group includes the ventral posterior, the ventral part (parvocellular) of the medial geniculate nucleus, the dorsal lateral geniculate nucleus (LGN), and the anterior thalamic nuclei, which receive somatosensory, auditory, visual, and mammillary afferents, respectively. There is evidence of layer V neocortical input for most of the other thalamic nuclei. These higher order nuclei have projections to higher order cortical regions (Clascá et al., 2012) and accumulating evidence points to the role of these nuclei in cognitive processes. See **Figure 2** for a schematic representation of thalamic nuclei at three anteroposterior levels of the rat thalamus, color-coded to indicate the first or higher order nature of each nucleus.

Modulators contribute a large proportion of the synapses that thalamic cells receive, their axonal terminals have thin and diffuse branches, and their terminals contain round small vesicles (they are called RS terminals, for their “Round” vesicles and “Small” size). Most RS terminals (the typical modulator morphology) contact the distal and intermediate parts of the dendrites. In cells reconstructed from thalamic first order nuclei, RS terminals form 40–80% of the synapses in distal and intermediate dendrites (Wilson et al., 1984; Raczkowski et al., 1988; Liu et al., 1995). The location of RS contacts far from the soma is consistent with them having a weak effect on spike generation. Still, in these studies the focus was on identifying terminal types, since RS terminals are likely to correspond to modulators. When modulators are identified by their neurotransmitter (reviewed below), additional terminal types and dendritic targets can be identified. For example, some modulator terminals (e.g., some cholinergic terminals in higher order nuclei) contact proximal dendrites, overlapping with driver synapses, and can be fairly large; and yet other modulators (serotonergic, noradrenergic, histaminergic) form “en passant” synapses, with little morphological specialization.

In first order thalamus, cortical layer VI and cholinergic inputs account for the majority of modulators. Each of these inputs contributes almost 50% of the RS terminals in the cat LGN (Erisir et al., 1997b). Also, after injections of retrograde tracer in first order thalamic nuclei, and staining for the tracer and for acetylcholine markers on the brainstem, the majority of cells are double-labeled (Mesulam et al., 1983; Hallanger et al., 1987; Lee et al., 1988; Steriade et al., 1988). The proportion of retrogradely labeled cholinergic brainstem cells was in the order of 70–85% when the retrograde tracer was injected in first order nuclei like the ventral posterior, LGN, and medial geniculate (Steriade et al., 1988), suggesting that most of the brainstem modulators to these nuclei originate in cholinergic cells.

In higher order nuclei, the overall number of modulator synapses is almost twice the number in first order nuclei (Van Horn and Sherman, 2007). This difference could result from an increased number of modulator axons sent to higher order nuclei, or it could reflect an increased number of synapses per axon. It could also indicate the existence of additional afferent centers providing extra modulator input to higher order cells. Consistent with the latter, the proportion of brainstem cells projecting to higher order nuclei that were cholinergic was roughly 60% in the cat ventral anterior, ventral lateral, and anterior ventral; 45% in the macaque lateral posterior and pulvinar nuclei; and as low as 28% in the cat mediodorsal (Steriade et al., 1988), although the same study found the fraction to be 82% in the cat lateral posterior, a proportion more similar to the first order thalamus. Only 25% of brainstem cells retrogradely labeled from tracer injections in the intralaminar centromedian and parafascicular nuclei were cholinergic (Paré et al., 1988). Overall, the evidence suggests that additional brainstem modulators (in addition to cholinergic) project to higher order nuclei.

Within the higher order nuclei, the midline and intralaminar groups are densely interconnected with executive areas (mPFC, basal ganglia). Additional higher order nuclei outside the midline and intralaminar project to executive regions (Vertes et al., 2014). For example, the anterior nuclei are highly interconnected with the cingulate and retrosplenial cortices, and with mPFC. The motor thalamus (ventral anterior, ventral lateral, and ventral medial nuclei) has projections to the basal ganglia and motor cortices. The anterior and motor groups have been studied mainly in the context of their roles in episodic memory and motor control, and little is known about their participation in executive function. For this reason, the second part of this review will focus on the midline and intralaminar nuclei.

## THALAMIC MODULATORS

### GLUTAMATE: LAYER VI CORTICOTHALAMIC MODULATORS

Layer VI afferents are the most studied of thalamic modulators. The evidence indicates that they form a complex network from layer VI sublaminae to first and higher order thalamic nuclei; they are topographically and functionally organized, and have an important role in sensory gain control.

#### Origin

Thalamic glutamatergic modulators originate in layer VI of neocortical areas (Jacobson and Trojanowski, 1975; Kaitz and Robertson, 1981; Kelly and Wong, 1981; Abramson and Chalupa, 1985; Giguere and Goldman-Rakic, 1988; Conley and Raczkowski, 1990; Ojima, 1994; Bourassa et al., 1995; Bourassa and Deschênes, 1995; Lévesque and Parent, 1998; Wang et al., 1999; Murphy et al., 2000; Kakei et al., 2001; Killackey and Sherman, 2003; Cappe et al., 2007; Briggs, 2010). Allocortical areas also send afferents to the thalamus (Price and Slotnick, 1983; Cornwall and Phillipson, 1988; Groenewegen, 1988; Risold et al., 1997; McKenna and Vertes, 2004; Cenquizca and Swanson, 2006; Varela et al., 2014), although their glutamatergic nature needs confirmation. Dekker and Kuypers (1976) reported the presence of small terminals in the thalamus after injection of tritiated aminoacids in hippocampus, which suggests that they are modulators, but the driver/modulator nature of hippocampo-thalamic projections remains to be investigated with modern techniques.****

In neocortex, about 30–50% of the pyramidal cells in layer VI project to the thalamus (Thomson, 2010), and the anatomy of corticothalamic projections suggests a high degree of topographic precision in the function of layer VI compared to other modulators (Murphy et al., 1999; Hazama et al., 2004). Layer VI also contains cortico-cortical projecting cells, but corticothalamic cells do not project to other cortical areas (Petrof et al., 2012). In addition, different subdivisions of layer VI project to first and higher order nuclei (Conley and Raczkowski, 1990; Bourassa et al., 1995; Bourassa and Deschênes, 1995; Killackey and Sherman, 2003), and the organization of projections increases in complexity in monkeys compared to rodents. In rats, pyramidal cells in the upper portion of layer VI of primary sensory cortices project to their corresponding first order nucleus (LGN, ventral posterior), while the lower layer VI projects to the higher order (posterior medial and lateral posterior nuclei). Axons from lower layer VI frequently branch to innervate both the first and higher order nuclei in rat (Bourassa et al., 1995; Bourassa and Deschênes, 1995). In prosimians (galago), lower layer VI cells do not branch and, instead, different subsets of cells provide input to the LGN and the pulvinar nuclei (Conley and Raczkowski, 1990). Of the three tiers of layer VI in macaques, only the upper and lower have corticothalamic projections. Each of these two sublaminae is part of a distinct functional network, with the upper layer targeting the magnocellular layers in LGN, as well as their cortical targets in layer IVCalpha. The lower layer VI sublamina projects to parvocellular LGN cells, as well as to their target, layer IVCbeta (Thomson, 2010; Briggs and Usrey, 2011). Whether functional classes in other nuclei are similarly organized in parallel circuits with layer VI remains an open question. It would be particularly interesting to investigate the functional organization in higher order cortical regions (mPFC, higher order sensory areas) of different animal groups, since these areas become relatively enlarged through evolution (Krubitzer and Seelke, 2012) and may gain in network complexity as well.

Higher order nuclei receive layer VI inputs from multiple cortical areas, and we know less about the specific sublaminae within layer VI that contribute afferents to higher order nuclei. One possibility is that layer VI feedback follows a similar pattern to that observed in first order. This would mean that corticothalamic afferents reciprocating a thalamocortical projection would have an upper layer VI component, whereas non-reciprocal corticothalamic projections would originate in lower layer VI. There is evidence of this arrangement in the somatosensory system, where the posterior medial nucleus receives input from upper layer VI of the non-barrel cortex to which it projects, and also from the lower layer VI of primary somatosensory cortex, a main target of the ventral posterior nucleus (Killackey and Sherman, 2003). Similar results have been reported for the macaque mediodorsal nucleus (Giguere and Goldman-Rakic, 1988), which receives upper layer VI input from mPFC as part of a reciprocal connection, but receives both upper and lower layer VI inputs from areas of the cingulate cortex that get only sparse mediodorsal afferents.

There is little information regarding the contributions from the contralateral hemisphere to the corticothalamic projections. Small terminals (potential layer VI projections) have been reported in the contralateral mediodorsal nucleus after unilateral tracer injections in mPFC (Négyessy et al., 1998). Contralateral projections were also demonstrated from the motor cortex to several motor, intralaminar, and somatosensory thalamic nuclei (Molinari et al., 1985; Alloway et al., 2008).

#### Local network organization

One of the key features that distinguish layer VI glutamatergic inputs from other glutamatergic inputs (e.g., layer V and non-cortical drivers) is the dendritic location of their synapses. Cortical modulators target mostly distal dendrites in both first and higher order nuclei (Robson, 1983; Kultas-Ilinsky and Ilinsky, 1991; Erisir et al., 1997a; Wang et al., 1999; Bartlett et al., 2000). In fact, the glutamatergic modulators contact the relay cells in more distal locations than other modulators (Erisir et al., 1997a).

The arborization pattern of individual axons is quite distinct, and *in vivo* results indicate that their geometrical shape is linked to the cell’s response properties. Individual axons from layer VI cells form terminal arbors with a plate-like (Ojima, 1994: ventral portion of the medial geniculate nucleus; Kakei et al., 2001: ventral anterior and lateral nuclei) or rod-like morphology (Bourassa et al., 1995: ventral posterior nucleus; Bourassa and Deschênes, 1995: LGN; Rockland, 1996: pulvinar nucleus). Bourassa et al. (1995) and Bourassa and Deschênes (1995) did not find a consistent arborization pattern in the posterior medial and lateral posterior nuclei. However, they did report that axonal plexuses were always in the horizontal plane in the lateral posterior nucleus, and showed examples of both rod and plate-like configurations. In the LGN, the orientation of the rod-like corticothalamic terminals correlates with the response properties of the cells of origin, with the orientation of the terminals being either parallel or perpendicular to the orientation preference of the cells of origin (Murphy et al., 1999); the functional correlates of these arborization patterns need to be tested in other first and in higher order nuclei.

#### *In vitro* results

Layer VI corticothalamic afferents have a direct depolarizing effect on relay cells (Scharfman et al., 1990; Reichova and Sherman, 2004; Miyata and Imoto, 2006), and an indirect hyperpolarizing effect through the activation of the thalamic reticular nucleus (TRN; Landisman and Connors, 2007; Lam and Sherman, 2010). The direct excitatory effect is mediated by both ionotropic and metabotropic receptors (mGluRs). Although with exceptions, group I mGluRs are postsynaptic, and groups II and III are localized in presynaptic terminals (Niswender and Conn, 2010). Of the two group I mGluRs, mGluR1 contributes to the corticothalamic excitatory postsynaptic potentials (EPSPs) in the LGN, ventral posterior, and posterior medial nuclei (McCormick and von Krosigk, 1992; Turner and Salt, 2000; Reichova and Sherman, 2004). Instead, groups II and III mediate presynaptic inhibition of corticothalamic responses, both the direct EPSP (Turner and Salt, 1999; Alexander and Godwin, 2005) and the inhibitory postsynaptic potentials evoked by the TRN (Salt and Turner, 1998; Turner and Salt, 2003). The inhibitory component from the TRN can also be diminished by cholinergic input (Lam and Sherman, 2010). Since activation of mGluRs increases with the intensity of stimulation, presynaptic inhibition through group II receptors could prevent over-activation or saturation of thalamic responses. Recent evidence indicates that mGluRs can also be active with relatively low frequency of stimulation, which brings up the possibility of their involvement throughout the response curve of relay cells (Viaene et al., 2013). Another property of layer VI corticothalamic synapses is that the direct response facilitates following repetitive stimulation. The facilitation is the result of both presynaptic and postsynaptic mechanisms (Miyata and Imoto, 2006; Sun and Beierlein, 2011), and it is stronger for the EPSPs evoked on relay compared to TRN cells (Alexander et al., 2006; Jurgens et al., 2012).

The activation of postsynaptic mGluRs is critical for one the proposed functions of corticothalamic modulators: switching the firing mode of relay cells (McCormick and von Krosigk, 1992; Godwin et al., 1996). Relay cells in the thalamus fire spikes in two modes, burst and tonic (Jahnsen and Llinás, 1984). In tonic mode, relay cells respond in a linear fashion to their inputs, while burst firing is non-linear but provides better detectability (Sherman, 2001). Burst firing relies on the activation of a transient (T-type), low threshold, calcium current. Changes in membrane potential determine the de-inactivation and activation state of the calcium channels responsible for burst firing (Jahnsen and Llinás, 1984; Gutierrez et al., 2001). De-inactivation of the T current takes about 100 ms, which falls within the timeframe of mGluRs responses. The relatively slow dynamics of mGluRs leads to slow changes in the membrane potential that can influence the firing mode. Thus, layer VI activation of a relay cell would make it more likely to fire spikes in tonic mode, facilitating faithful signal transmission (Sherman, 2001).

#### Systems level

Most of the *in vivo* studies on corticothalamic projections have been done in the visual system in anesthetized preparations (recent reviews include Cudeiro and Sillito, 2006; Sillito et al., 2006; Briggs and Usrey, 2011), and only recently in awake animals (Olsen et al., 2012; Pais-Vieira et al., 2013). In the visual system, layer VI corticothalamic projections can influence center-surround strength without changing the spatial selectivity of receptive fields (Rivadulla et al., 2002; Jones et al., 2012). An important aspect of the corticothalamic input is that it is topographically and functionally organized, meaning that specific functional types of LGN cells (X, Y, W or parvocellular, magnocellular, koniocellular) will be influenced by layer VI cells with similar response properties. However, the effect on the firing rate of relay cells is reversed depending on the overlap of on–off receptive field regions. For example, an on-center relay cell with a receptive field overlapping with the “off” portion of a corticothalamic receptive field, would receive an excitatory influence from cortex, whereas if the overlapping fields were of the same sign, the influence would be inhibitory (Wang et al., 2006). Topographically organized effects are also observed in the somatosensory system, where activation of layer VI cells produced opposite effects on simultaneously recorded neighboring thalamic barreloids. During layer VI activation, cells in non-aligned thalamic barreloids were suppressed and less selective to preferred whisker stimulation. Instead, during activation of layer VI, responses in the topographically aligned barreloid were selectively increased to preferred whisker stimulation, leading to an increase in spatial tuning selectivity (Temereanca and Simons, 2004). Enhanced responses were also seen in thalamic barreloids after activation of topographically aligned regions in motor cortex, which could contribute to sensory gating and anticipatory responses in cortex and thalamus during active whisking (Lee et al., 2008; Pais-Vieira et al., 2013).

**FIGURE 3:**
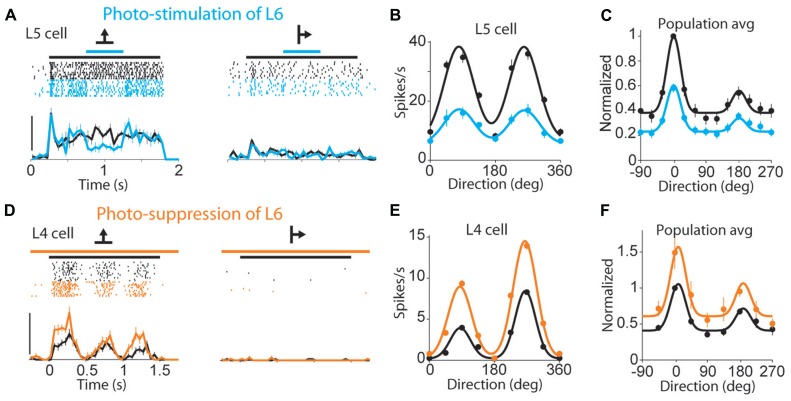
**Layer VI contributes to gain control in mouse visual cortex. (A)** Response of a layer V cell (spike rasters and peri-stimulus histograms) to visual stimuli with and without photostimulation of layer VI; black line above raster indicates stimulus presentation, blue indicates the time of optogenetic activation of layer VI. Visual stimuli were full-field gratings drifting in different directions (arrows); scale bar, 40 spikes/s. **(B)** Tuning curves for the cell in **(A)**, including the responses to nine stimulus directions, with (blue) and without (black) photostimulation of layer VI. **(C)** Population tuning curve with (blue) and without (black) photostimulation of layer VI; the population tuning curve was generated by first circularly shifting the stimulus direction for each unit so that the maximal response occurred at 0°. The responses were then normalized to this peak response and averaged (*n* = 55 units). **(D–F)** same as **(A–C)** but during photosuppression of layer VI, and using a cell from layer IV as example; scale bar in **(D)** 50 spikes/s; population tuning curve in **(F)** is the average of *n* = 52 units. (Reprinted from Olsen et al., 2012, with permission from Macmillan Publishers.)

The results from sensory systems demonstrate contributions to sensory processing, but corticothalamic inputs are found in every thalamic nucleus, which implies functions beyond specific sensory modalities. Layer VI cells receive input from all cortical layers and could serve to integrate processed cortical information with the direct input from the thalamus (Thomson, 2010). On the other hand, the effect of corticothalamic inputs on membrane potential points to a gain control system. There is evidence in support of the gain control hypothesis in the mouse visual cortex (Olsen et al., 2012), in which optogenetic manipulation (activation and inhibition) of layer VI scaled the tuning curves of cortical cells up and down without changes in response selectivity (**Figure 3**). Stimulation of layer VI linearly reduced cortical responses to the presentation of full-field gratings moving in different directions (**Figures 3A–C**), while inhibition of layer VI increased cortical responses (**Figures 3D–F**). This linear modification of the cortical tuning curves was found to result from the effect of layer VI on other cortical layers and on thalamic LGN cells. However, the effect on tuning curves was not tested in LGN, and the role of layer VI on gain control deserves further exploration at the thalamic level. In particular, although other modulators have an effect on membrane potential and could influence thalamic gain, the topographic and functional organization of the corticothalamic projection suggests that layer VI provides a more precise control than other modulators. Along these lines, corticothalamic projections could carry out topographically specific, top-down gain control in sensory nuclei as a function of ongoing neocortical processing. It has also been suggested that they could implement predictive modulation (Sillito et al., 2006) in expectation of stimulus arrival or stimulus changes, such as when processing a moving stimulus. Future experiments should test these hypotheses, and step beyond sensory cortices to explore the role of layer VI in other thalamocortical networks.

### ACETYLCHOLINE

Cholinergic systems have been broadly involved in state regulation (sleep–wake cycle, attention) and may contribute to state dependent changes in information routing in neocortex. The thalamus receives cholinergic input from a variety of sources that preferentially innervate higher order nuclei and, through these nuclei, could contribute to cholinergic mediated modulation in neocortex. In the thalamus, cholinergic terminals can have large synaptic boutons (with potentially strong postsynaptic effects), and the effect on relay cells can be circuit specific, determined by the cell’s projection target.

#### Origin

Cholinergic input to the thalamus originates mainly in the pedunculopontine (PPT) and the laterodorsal tegmental (LDT) nuclei (Saper and Loewy, 1980; Mesulam et al., 1983; Sofroniew et al., 1985; Woolf and Butcher, 1986). Cholinergic neurons in the PPT and LDT are intermingled with non-cholinergic neurons but, after injection of retrograde tracers in the thalamus, most of the retrograde tracer is found in choline acetyltransferase positive neurons, suggesting that the non-cholinergic cells project sparsely to the thalamus (Mesulam et al., 1983; Sofroniew et al., 1985). Besides the PPT and LDT afferents, some thalamic nuclei (the mediodorsal, anterior ventral, anterior medial, and anterior intralaminar nuclei) receive cholinergic projections from the basal forebrain (Hallanger et al., 1987; Parent et al., 1988; Steriade et al., 1988; Heckers et al., 1992; Gritti et al., 1998), a region otherwise projecting to cortical areas and to the TRN (Saper, 1984; Hallanger et al., 1987). The parabigeminal nuclei provide additional cholinergic input to the LGN of cats and monkeys, a projection that is both ipsi- and contralateral in cats and strictly contralateral in the tree shrew (De Lima and Singer, 1987; Fitzpatrick et al., 1988, 1989; Smith et al., 1988; Bickford et al., 2000). Lastly, cholinergic neurons from the entopeduncular nucleus (Kha et al., 2000) and *substantia nigra* (*pars reticulata*; Kha et al., 2001) send axons to the rat ventral lateral and ventral medial nuclei, both part of the motor thalamus. Within the diencephalon, the medial habenula contains cholinergic neurons (Levey et al., 1987; Heckers et al., 1992), but its efferents appear to be directed outside the dorsal thalamus (Vincent et al., 1980).

#### Local network organization

PPT and LDT cholinergic projections have preferential targets within the thalamus. Sensory nuclei (LGN, ventral posterior, and the medial geniculate nuclei) receive most of their cholinergic afferents from PPT, whereas higher order nuclei and the anterior group have a LDT component (Woolf and Butcher, 1986; Hallanger et al., 1987; Smith et al., 1988; Steriade et al., 1988). This additional LDT innervation may contribute to the higher density of cholinergic fibers observed in some higher order compared to first order nuclei (Parent and Descarries, 2008).

Within the higher order group, the mediodorsal, the lateral posterior, ventral anterior, ventral lateral, laterodorsal, and posterior nuclei receive a substantial fraction of their cholinergic input from LDT. The two latter nuclei receive about two thirds of their brainstem cholinergic input from PPT and a third from LDT. Within the intralaminar, the central lateral seems to be primarily targeted by PPT, while the central medial has a large component from LDT (Woolf and Butcher, 1986; Hallanger et al., 1987). Anterograde tracers have also demonstrated LDT projections to the midline nuclei (Kuroda and Price, 1991); however, the relative contribution of PPT and LDT to the midline cholinergic innervation was not addressed in this study.

At least some of the cholinergic brainstem axons have collaterals that innervate more than one nucleus in the dorsal thalamus (Uhlrich et al., 1988; Shiromani et al., 1990; Bolton et al., 1993), and can innervate the TRN as well (Spreafico et al., 1993). In some cases, the axons remain within nuclei of a particular sensory modality; e.g., the collaterals that innervate the LGN, lateral posterior, and pulvinar nuclei in cat (Uhlrich et al., 1988). There are other patterns of collateral projections, e.g., those that branch into several of the midline nuclei, or to midline and intralaminar (Bolton et al., 1993), or to LGN and intralaminar nuclei (Shiromani et al., 1990). More localized projections have been documented in the visual thalamus. Here, some axons terminate only in the LGN or only in the lateral posterior and pulvinar nuclei. Axons within the LGN distribute terminals across laminae or inside individual laminae (Uhlrich et al., 1988). It should be noted that in this study axons were not identified as cholinergic; however, results from retrograde tracer studies (see introduction) suggest that most or all of the reconstructed axons were cholinergic.

Cholinergic cells projecting to the thalamus can have branches to extra-thalamic regions as well. PPT projects both to the LGN and to the superior colliculus (Billet et al., 1999). Similarly, subsets of cells in PPT and LDT that project to the thalamus also project to the pontine reticular formation (Semba et al., 1990) and to the basal forebrain (Losier and Semba, 1993). The collaterals of cholinergic projections may contribute to the multi-regional coordination of state changes brought about by this system.

The ultrastructure of cholinergic terminals has been studied in a few first order – LGN, ventral posterior –, and higher order – anterior ventral, mediodorsal, parafascicular – nuclei (Hallanger et al., 1990; Kuroda and Price, 1991; Parent and Descarries, 2008). One feature of the LGN PPT terminals is that they contain the enzyme nitric oxide synthase (Cucchiaro et al., 1988; Hallanger et al., 1990; Bickford et al., 1993; Erisir et al., 1997a). In fact, cholinergic afferents may be the main, or even the sole, source of nitric oxide in the thalamus; although some serotonergic cells in the raphe express nitric oxide synthase, they do not project to the thalamus (Simpson et al., 2003). In the LGN, PPT terminals form asymmetric synapses on proximal and distal dendrites of relay cells, often in the vicinity of driver synapses, and occasionally in the soma. Compared to the LGN, the cholinergic terminals in the ventral posterior nucleus are sparser, smaller, and they establish asymmetric synapses on small dendrites (farther from the soma; Hallanger et al., 1990). The main difference between the ultrastructure of cholinergic terminals in first order and higher order nuclei is the much larger size in higher order. In both the mediodorsal and parafascicular nuclei, they can reach more than 2 μm (Hallanger et al., 1990; Kuroda and Price, 1991). In the mediodorsal nucleus, 90% of LDT boutons were larger than 1 μm, which is a size range more typical of drivers. In the ventral anterior nucleus, cholinergic terminals were less than 1 μm, but still larger on average than the terminals reported in the LGN and ventral posterior nuclei in the same preparation, suggesting a stronger effect on cells of higher order nuclei. The cholinergic terminals in the anterior ventral nucleus contacted dendrites of various sizes (often small dendrites and rarely somas), and they made occasional symmetric synapses in addition to the most common asymmetric contacts (Hallanger et al., 1990). The presence of nitric oxide synthase was not tested in higher order nuclei.

The larger cholinergic terminal size and fiber density in higher order nuclei may result in stronger postsynaptic effects on higher order compared to first order relay cells, something that can have important implications in cortical regions. As an example, association neocortical areas (those receiving afferents from higher order nuclei) present greater attentional modulation than primary cortical regions (Bender and Youakim, 2001; Maunsell and Cook, 2002), a function in which the cholinergic system may be involved. The attentional modulation observed in neocortex could reflect modulation at the thalamic level. Indeed, the evidence suggests that higher order nuclei, such as the pulvinar nucleus, have stronger attentional modulation than first order like the LGN (Bender and Youakim, 2001), and contribute to corticocortical synchronization during attentional tasks (Saalmann et al., 2012). Future manipulation experiments of higher order nuclei while observing the effect on attentional modulation in thalamus and cortex simultaneously, will help clarify the causal contribution of the thalamus to attentional modulation in cortical regions. 

Another open question is the origin of the large cholinergic terminals. Higher order nuclei receive a substantial projection from the LDT, and one possibility is that LDT axons provide the larger terminals observed in the thalamus. A further point related to the terminal size is that large terminal size is commonly associated with drivers and not modulators. Cholinergic afferents with large terminals could have a strong effect on spike generation probability on higher order cells (e.g., in the mediodorsal and parafascicular nuclei) because, in addition to having a large size, cholinergic terminals in these cells contact dendritic regions that are close to the soma. Both the lateral mediodorsal nucleus and LDT have been suggested to participate in oculomotor control (Kuroda and Price, 1991) and it is possible that the LDT projection represents a driver input to the mediodorsal nucleus.

#### *In vitro* and systems level

**FIGURE 4:**
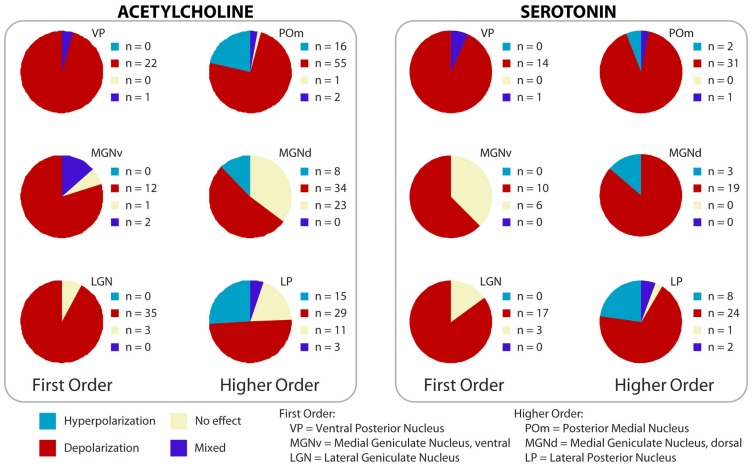
**Effect of cholinergic and serotonergic agonists on first and higher order nuclei**. Summary of the effects on membrane potential across thalamic nuclei from whole-cell patch clamp experiments in rat slices; data are color-coded according to the overall effect on excitability. Hyperpolarization was only found in higher order nuclei. First order nuclei tested: VP, MGNv, LGN; higher order tested: POm, MGNd, LP. (Modified from Varela and Sherman, 2007, 2009; with permission from the American Physiological Society and from Oxford University Press.)

Cholinergic activation depolarizes the majority of thalamic cells (Sillito et al., 1983; Francesconi et al., 1988; Curró Dossi et al., 1991), although some relay cells, as well as thalamic interneurons, are hyperpolarized by cholinergic agonists (McCormick and Prince, 1986; McCormick and Pape, 1988; Hu et al., 1989; Murphy et al., 1994; Zhu and Heggelund, 2001; Varela and Sherman, 2007). In general, relay cells that are hyperpolarized by acetylcholine are in higher order nuclei (MacLeod et al., 1984; Mooney et al., 2004; Varela and Sherman, 2007; Beatty et al., 2009). Interestingly, at least in one higher order nucleus (the parafascicular), the sign of the cholinergic effect correlates with the projection target of the cell. Relay cells projecting to neocortex are depolarized by cholinergic agonists, whereas those projecting to striatum are inhibited (Beatty et al., 2009). This result has key implications for the function of thalamostriatal projections in behavioral flexibility, and will be discussed in the second part of this review. It also raises the possibility that the depolarizing or hyperpolarizing effect of modulators may be pathway specific in other nuclei; given the variety of modulator effects in higher order nuclei (**Figure 4**), the correlation between modulator effect and projection target needs to be tested for pathways from these nuclei.

Mixed responses, in which a hyperpolarization is followed by depolarization, have also been reported. This combined response was observed in the lateral posterior nucleus, in interneurons of the LGN (Zhu and Heggelund, 2001), and in a subset of cells of the ventral medial nucleus (MacLeod et al., 1984). It was also reported in about half of the cells in the guinea pig lateral and medial geniculate nuclei (McCormick and Prince, 1987; McCormick, 1992), and could represent species differences, with depolarization being the most common response in rat first order nuclei.

Overall, cholinergic-evoked depolarization (whether by itself or as part of a mixed response) is mediated by ionotropic and muscarinic (M1, M3) receptors (Zhu and Uhlrich, 1997, 1998; Mooney et al., 2004; Varela and Sherman, 2007), whereas the M2 muscarinic receptor is responsible for the hyperpolarization of GABAergic cells (McCormick and Prince, 1986; Zhu and Heggelund, 2001).

Aside from the effect on membrane potential, other effects of acetylcholine at the thalamic level have not been extensively studied. Results outside the thalamus suggest that there is much to be explored regarding the functions of the cholinergic system in the thalamus (Picciotto et al., 2012), especially in behaving animals. In the slice preparation, acetylcholine affects neurotransmitter release and synaptic strength in intracortical and thalamocortical synapses (Favero et al., 2012), changes that can be important during the implementation of bottom-up and top-down attentional regulation (Varela, 2013) and can only be studied in the behaving animal. In addition, results from a head-restrained preparation show that the effects on membrane potential observed in the slice may vary *in vivo* throughout the sleep–wake cycle. Iontophoretic application of cholinergic agonists in the LGN depolarized cells during wakefulness, as expected from the *in vitro* results, but had heterogeneous effects during slow-wave sleep (Marks and Roffwarg, 1989). Lastly, cholinergic activation enhances thalamocortical information transmission through nicotinic receptors located along the axons of the thalamocortical pathway (Kawai et al., 2007), a result that remains to be investigated in thalamic projections to other targets, like the basal ganglia and hippocampus.

### SEROTONIN

Serotonergic afferents to the thalamus have not received much attention, in spite of the critical involvement of serotonin in the control of the sleep–wake cycle and in disorders like depression (Monti, 2011; Kupfer et al., 2012). In the thalamus, serotonergic afferents target preferentially higher order nuclei, where they have heterogeneous effects on membrane potential and could evoke changes in firing mode throughout the sleep–wake cycle.

#### Origin and local network organization

The serotonergic axons innervating the thalamus have their origin in the medial and lateral divisions of the dorsal raphe (De Lima and Singer, 1987; Vertes, 1991; Gonzalo-Ruiz et al., 1995; Vertes et al., 1999, 2010; Kirifides et al., 2001), and in the median raphe (Gonzalo-Ruiz et al., 1995; Vertes et al., 1999). The projections do not always overlap; for example, the median raphe projects most heavily to the lateral mediodorsal nucleus, while the medial mediodorsal nucleus receives serotonergic input from the dorsal raphe (Groenewegen, 1988).

Just like with the cholinergic input, the distribution of serotonergic fibers within the thalamus is not uniform. The preferential targets are the midline and intralaminar nuclei, and, more generally, the higher order nuclei. The rest of the dorsal thalamus receives sparse innervation with the exception of the LGN (Cropper et al., 1984; Lavoie and Parent, 1991; Vertes, 1991; Vertes et al., 1999, 2010). There is some evidence of local differences in innervation density within nuclei. The heaviest serotonergic innervation in the LGN is generally found in structures receiving input from W-ganglion cells (Ueda and Sano, 1986; Mize and Payne, 1987; Fitzpatrick et al., 1989), although others have found uniform innervation across the LGN and lateral posterior and pulvinar nuclei (Morrison and Foote, 1986).

Serotonergic afferents form asymmetric synapses along the dendrites (distal and proximal) of thalamic cells (Pasik et al., 1988; Liu and Jones, 1991). They also form atypical contacts (Liu and Jones, 1991), meaning that they do not present all the morphological specializations of a synapse, only a close membrane apposition.

#### *In vitro* and systems level

Serotonin depolarizes thalamic cells in first order nuclei, such as the LGN, the ventral portion of the medial geniculate, the ventral posterior, and the anterior dorsal nuclei (Pape and McCormick, 1989; McCormick and Pape, 1990; Chapin and Andrade, 2001a; Monckton and McCormick, 2002). The depolarization results, at least in part, from changes in the voltage-dependence of the hyperpolarization-activated current, I_h_ (Pape and McCormick, 1989; McCormick and Pape, 1990; Chapin and Andrade, 2001b; Monckton and McCormick, 2002). Subsets of cells in higher order nuclei are either depolarized or hyperpolarized, and the proportion of cells that show one or the other response varies between species (Monckton and McCormick, 2002; Varela and Sherman, 2009). When compared in the same preparation, the depolarization is much stronger in higher order than in first order areas (Varela and Sherman, 2009), consistent with the denser innervation in those nuclei. Overall, both acetylcholine and serotonin inhibit a subset of cells specifically in higher order nuclei, while the effect is mostly depolarizing in first order (**Figure 4**). The inhibition of cells in higher order means that, when active, these modulators could switch some cells to burst mode, which can contribute to the finding of more bursting in higher compared to first order nuclei (Ramcharan et al., 2005). In addition to the effect on the membrane potential, there is evidence that serotonin affects the response properties of some relay cells. Cells in the midline and intralaminar nuclei have a strong slow afterhyperpolarization (sAHP) that can last several seconds after a train of spikes. Serotonin depolarizes cells in these nuclei and inhibits the sAHP through 5-HT-7 receptors (Goaillard and Vincent, 2002).

There is little information from *in vivo* preparations on the role of serotonin on thalamic function. Activation of the dorsal raphe nucleus was reported to inhibit LGN cells in the anesthetized preparation (Kayama et al., 1989). However, this was observed after several seconds of stimulation, and could result from changes in synaptic plasticity somewhere else in the brain (Lesch and Waider, 2012). Another report in the anesthetized preparation (Grasso et al., 2006), found that serotonergic agonists infused in the motor thalamus (ventral anterior and ventral lateral nuclei) produced an inhibition of the discharge of these cells, consistent with the *in vitro* findings in higher order nuclei. The systems level approach to serotonergic function in the thalamus remains essentially uninvestigated. The study of serotonin outside the thalamus hints at critical roles for this neurotransmitter; from synapse development and plasticity to the learning of fear responses (Lesch and Waider, 2012). Future experiments should characterize the effect of serotonergic afferents on sensory responses, and on the response mode of thalamic cells across sleep states. Much like brainstem cholinergic centers, cells in the raphe change their activity as a function of state (Monti, 2011). Many of the raphe cells are REM-OFF, suggesting a reduction in serotonergic tone in the thalamus during REM, a reduction that can selectively affect the firing mode of higher order cells. An intriguing idea is that changes in firing mode in higher order nuclei could contribute to the selective activation of higher order cortical areas during REM, an activation that is thought to underlie dreaming (Hobson et al., 1998).

### NORADRENALINE

Like with serotonin, the studies of noradrenergic modulation in the thalamus are fairly limited and much remains to be investigated. Recent evidence offers important cues that could instigate further research on this neurotransmitter; these results suggest a role of thalamic noradrenaline in sensory gating and in certain motor and executive disorders.

#### Origin and local network organization

The cells that provide noradrenergic afferents to the brain are located in the locus coeruleus (LC) and in the brainstem reticular formation. The thalamus receives its noradrenergic input mostly from cells in the LC – many of which also contain galanin (Simpson et al., 1997). Additional projections have been reported for the midline paraventricular nucleus from the A5 noradrenergic region in the brainstem (Swanson and Hartman, 1975; Morrison and Foote, 1986; Byrum and Guyenet, 1987; De Lima and Singer, 1987; Simpson et al., 1997; Vogt et al., 2008).

As with acetylcholine and serotonin, there are regional differences in the innervation of thalamic nuclei. For example, the LGN is virtually free of noradrenergic fibers, while the lateral posterior and pulvinar nuclei are densely innervated (Morrison and Foote, 1986). In the somatosensory thalamus, noradrenergic innervation is denser in the posterior medial nucleus (higher order) compared to the ventral posterior nucleus (Simpson et al., 1999). Therefore, similar to other modulators, the results in the sensory thalamus point to a more prominent role of noradrenaline in higher compared to first order nuclei. However, the limited evidence from the midline and intralaminar nuclei suggests that they receive sparse noradrenergic innervation, except for the midline paraventricular nucleus (Swanson and Hartman, 1975). Regarding ultrastructure, noradrenergic terminals in the thalamus are small, and, like serotonergic terminals, do not seem to form well differentiated synapses (Nothias et al., 1988).

#### *In vitro* and systems level

Noradrenaline applied *in vitro* to the LGN, medial geniculate, TRN, anterior ventral, and the paratenial nuclei, evoked a slow depolarization, which in turn reduced burst firing and promoted tonic activity (McCormick and Prince, 1988). The authors found that the depolarization was caused by a decrease in a potassium leak current and by changes in the voltage sensitivity of the I_h_ current. The I_h_ current could then remain active at resting membrane potentials and make it more difficult for cells to switch to burst mode (Pape and McCormick, 1989; McCormick and Pape, 1990). The effect of noradrenaline on the response properties of relay cells was tested in paratenial neurons, in which noradrenaline reduced the sAHP and decreased spike frequency adaptation (McCormick and Prince, 1988).

*In vivo*, in the anesthetized preparation, iontophoretic application of noradrenergic agonists inhibits thalamic cells in the motor thalamus (ventral anterior and ventral medial nuclei; Grasso et al., 2006). The sign of the effect is the opposite of that found by *in vitro* experiments, where depolarization was common. More research is needed to clarify if the different results indicate the variability of the responses across thalamic nuclei, or an effect of the anesthesia. Evidence from the awake preparation suggests that, although depolarization predominates in the somatosensory thalamus, inhibitory responses are fairly common too. Responses to whisker stimulation increased in most cells of the ventral posterior nucleus during stimulation of the LC, although between 20% (Moxon et al., 2007) and almost 40% (Devilbiss and Waterhouse, 2011) of the cells showed a suppression of their response. In particular, phasic stimulation of the LC had a permissive or “gating” effect in some cells, facilitating the response to a stimulus that the cell would otherwise not respond to in the absence of LC stimulation. Stimulation of the LC also enhanced the synchronization of sensory responses between simultaneously recorded cells in the ventral posterior nucleus, with potential implications on temporal summation at the cortical level (Devilbiss and Waterhouse, 2011). Furthermore, noradrenaline changed the synaptic strength of intracortical and thalamocortical synapses in the slice preparation (Favero et al., 2012). In this study, noradrenaline facilitated thalamocortical relative to intracortical transmission in the input layers of cortex, a result that has implications for the routing of external vs. internal information during the sleep–wake cycle (Varela, 2013).

Aside from the effects on sensory gating, recent evidence suggests the involvement of thalamic noradrenaline modulation in executive and motor disorders. Infusion of noradrenergic agonists (but not serotonin) in the mediodorsal nucleus disrupts prepulse inhibition; prepulse inhibition paradigms are used as indicators of sensorimotor gating disruption in neuropsychiatric disorders, and it was suggested that noradrenergic activation in the mediodorsal nucleus reproduces some of the sensorimotor gating deficits observed in these disorders (Alsene et al., 2011). Likewise, noradrenaline may be critical for the normal function of the motor thalamus, which is suggested by the specific decrease of this neurotransmitter in the motor thalamus of the symptomatic MPTP (methyl-phenyl-tetrahydropyridine) primate model of Parkinson disease (Pifl et al., 2013). Overall, the available evidence indicates that noradrenergic modulation in the thalamus can influence sensory responses and, potentially, has considerable clinical relevance.

### DOPAMINE

Dopamine is one of the thalamic modulators with more direct involvement in disease. The degeneration of dopaminergic cells in the *substantia nigra pars compacta* links this modulator to the pathogenesis of Parkinson disease. In addition, the role of dopaminergic cells from the ventral tegmental area (VTA) in reward signaling is thought to contribute to addiction, and to the symptomatology of disorders such as schizophrenia and depression. The thalamus does not receive strong dopaminergic innervation from the *substantia nigra*, but it gets dopamine afferents from the VTA and additional mesencephalic and diencephalic regions. Also, dopaminergic terminals are often near thalamic terminals at their targets (e.g., neocortex, striatum), indicating that at least some of the thalamic dopaminergic modulation may occur not at the soma, but at the terminal site.

#### Origin and local network organization

There is a wide range of brain areas, particularly in the primate, that provide dopaminergic input to the thalamus, including the hypothalamus, zona incerta, the VTA, the periaqueductal gray, and the lateral parabrachial nucleus, all of which project bilaterally to most nuclei of the macaque thalamus (Hughes and Mullikin, 1984; Sánchez-González et al., 2005). Dopaminergic projections to the thalamus from the *substantia nigra* are minimal, although there are non-dopaminergic projections from this region (Kuroda and Price, 1991; Sánchez-González et al., 2005; Melchitzky et al., 2006; Kusnoor et al., 2012). Some afferents, like those from the VTA, project broadly across the thalamus, whereas others have restricted projections, like those from the hypothalamus and zona incerta, which have dense projections to the midline thalamus. Most of the projections to the midline do not express the dopamine transporter, and it has been suggested that the absence of the transporter could make the effect of dopamine less time and spatially restricted in these nuclei. The absence of the dopamine transporter has clinical implications as well, because this transporter is the point of action of drugs (amphetamines) and toxins (MPTP), suggesting that the midline dopaminergic afferents would be relatively protected against these substances compared to other nuclei (Sánchez-González et al., 2005).

There are important species differences in the density of thalamic dopaminergic innervation, with the primate thalamus having substantially higher densities compared to the rat (García-Cabezas et al., 2009). Dopaminergic fibers in the thalamus of primates often display higher densities than in cortex, and the density is highest in the motor and midline thalamus, and the lateral posterior nucleus (Sánchez-González et al., 2005); the lowest densities are found in sensory first order nuclei (LGN, medial geniculate, and ventral posterior nuclei). In primates, dopaminergic terminals contact the presynaptic dendrites of thalamic interneurons, raising the possibility that the denser dopaminergic innervation in primates is related to the increased number of interneurons in these animals (García-Cabezas et al., 2009).

#### *In vitro* and systems level

Outside of the thalamus, two types of dopaminergic receptors, D1 and D2, are often segregated in functional circuits, something that has yet to be explored in detail in the thalamus. Along these lines, D2 receptors are highly expressed in midline and intralaminar nuclei (Rieck et al., 2004; Piggott et al., 2007), and D1–D2 receptors mediate different effects on membrane potential in different nuclei. D1 mediates the depolarization of rat LGN cells in slices (Govindaiah and Cox, 2005), and D2 the hyperpolarization of most cells in the mediodorsal nucleus (Lavin and Grace, 1998). Furthermore, in the mediodorsal nucleus, D2 can influence the cells response properties, by facilitating the occurrence of low threshold burst spikes and increasing the sAHP (Lavin and Grace, 1998). Other dopaminergic receptors are present in the presynaptic terminals of thalamic afferents; for example, D4 can presynaptically and selectively decrease the inhibitory input from the *globus pallidus* to the TRN (Govindaiah et al., 2010).

*In vivo*, the results of iontophoretic application of dopamine were found to be dose-dependent, with dopamine facilitating visual responses at low doses and inhibiting responses at higher doses (Albrecht et al., 1996; Zhao et al., 2001, 2002). The inhibition at higher doses could result from the activation of local interneurons or TRN cells. Iontophoresis of D1 agonists suppressed visual responses in these studies, something in contrast to the depolarization seen in slices (Govindaiah and Cox, 2005); the use of more selective agonists and antagonists could help resolve the differences and characterize the effect of dopamine in sensory evoked responses.

The relatively weak dopaminergic innervation of the rat thalamus may have discouraged research on the function of this modulator at the thalamic level. However, the importance of dopamine modulation on thalamic function should not be underestimated. First, the dramatic increase in dopaminergic innervation in the primate thalamus compared to the rodent thalamus points to the evolutionary relevance of this system; it also suggests that dopamine may be specifically relevant for those functions that gain in importance through evolution, such as higher order cognitive functions. And, second, dopaminergic and thalamic synapses often converge on the same postsynaptic targets outside of the thalamus (Kuroda et al., 1996), suggesting that thalamic dopaminergic modulation may be more likely to occur at the level of thalamic terminals than at the soma.

### HISTAMINE

Very little is known about the modulator functions of histamine in the thalamus, with most of the evidence coming from studies in the LGN. The activity of histaminergic cells varies across the sleep–wake cycle suggesting that, similar to serotonin, noradrenaline, and acetylcholine, this modulator may be involved in the regulation of general changes of activity across states of vigilance. However, the effect of histamine on the excitability of thalamic cells, and the selective modulation of thalamostriatal terminals by histamine suggest more complex functions that need to be investigated.

#### Origin and local network organization

Histaminergic input arises from the tuberomammillary nucleus of the hypothalamus (Manning et al., 1996; Blandina et al., 2012). In the cat LGN, histaminergic fibers have a preference for zones innervated by the W-cell system (Uhlrich et al., 1993), although their distribution is more homogeneous in the macaque LGN (Manning et al., 1996; Wilson et al., 1999). No clear synaptic contacts were observed, only *en passant* swellings, which hint to a diffuse modulation mechanism (Uhlrich et al., 1993; Wilson et al., 1999).

#### *In vitro* and systems level

*In vitro*, LGN cells are depolarized by histamine. The response has two components, the main one being an increase in input resistance mediated by H1 receptors. The second component is a smaller depolarization, which is observed after blockade of H1 receptors, is mediated by H2 receptors, and is associated with a decrease in input resistance (McCormick and Williamson, 1991). These *in vitro* results in the LGN are consistent with the effect of activating the tuberomammillary region *in vivo*, which results in increased firing in LGN cells, with no change of their spatial frequency tuning (Uhlrich et al., 2002). Conversely, a study testing iontophoretically applied histamine in the anterior and intralaminar nuclei found an inhibition of baseline firing (Sittig and Davidowa, 2001). More research is needed to characterize the effects of histamine across the thalamus and identify the receptors that mediate the responses in different nuclei. There are additional histaminergic receptors in thalamic cells (H3, H4), but evidence of their function is limited (Strakhova et al., 2009). In particular, H3 presynaptic receptors could be important in the modulation of thalamostriatal terminals, where they are expressed; these receptors selectively facilitate thalamostriatal – and not corticostriatal – synapses during repetitive stimulation (Ellender et al., 2011).

Cells of the tuberomammilary nucleus are only active during wakefulness and their degree of activation correlates with alertness levels (Takahashi et al., 2006), suggesting that its function in the thalamus may relate to attentional levels and state related changes through the sleep–wake cycle.

## THALAMIC MODULATORS AND EXECUTIVE FUNCTION

The data reviewed in the previous section suggests that modulators contribute to the function of virtually all thalamic nuclei and may be critical in higher order nuclei. These nuclei receive a higher proportion of modulators than first order, have cell populations with heterogeneous responses to modulators, and are interconnected with brain regions that are themselves under strong modulator control.

One feature that characterizes higher order thalamic nuclei is the complexity of their projections. Whereas sensory nuclei have relatively confined projection targets within neocortex, higher order nuclei project to multiple regions within and outside of neocortex. Targets include the basal ganglia, hippocampus, hypothalamus, and amygdala. Among them, mPFC and the striatum have been identified as key structures in the control of executive function. Although a few other thalamic nuclei project to these two areas, the following section will focus on the modulation of two groups of nuclei that have strong connections with mPFC and the striatum: the midline and the intralaminar groups (Hoover and Vertes, 2007; Galvan and Smith, 2011). The midline group includes, ventrally, the reuniens and rhomboid nuclei, and, more dorsally, the paratenial, paraventricular, and mediodorsal nuclei. This group is defined primarily by its position along the midline of the thalamus, and the mediodorsal, the reuniens, and the paratenial nuclei also originate from the same pronuclear mass during development (Jones, 2007). The intralaminar nuclei follow an anteroposterior axis, with the rostral part including the central lateral, paracentral, and central medial nuclei. The parafascicular nucleus, together with the centromedian nucleus in primates, constitute the caudal components of the intralaminar group and are the main source of thalamic input to the striatum (Galvan and Smith, 2011). The midline and intralaminar nuclei have other projection targets (e.g., the hippocampus and amygdala), and modulators in these nuclei can therefore influence networks beyond those directly involved in executive function.

Many open questions remain regarding the function of the midline and intralaminar nuclei. In most cases we lack even basic information, such as which area (or areas) drives these nuclei, or what are the receptive field properties of their cells. Nonetheless, some of the nuclei have been implicated in functional loops in which modulators play a critical role. I will review those here.

One of the first functions proposed for the midline and intralaminar nuclei, and in which modulators are involved, was state maintenance. Moruzzi and Magoun’s (1949) classic study raised the possibility that the intralaminar nuclei could mediate the effect of the reticular activating system on the neocortex during wakefulness. The cortical projections of the midline and intralaminar nuclei, which innervate the superficial layers of multiple regions, gave support to the idea that the reticular activating system could influence neocortex through the activation of the midline-intralaminar thalamus. This is consistent with the disruption of consciousness that follows damage to this thalamic region in humans (Llinás et al., 1998), as well as with the improvement that follows deep brain stimulation of the intralaminar nuclei in patients in the minimally conscious state (Schiff et al., 2007). Likewise, brainstem cholinergic and monoaminergic regions promote wakefulness through their effect on multiple regions (Lee and Dan, 2012), and they innervate midline and intralaminar nuclei extensively (reviewed above). On the other hand, the traditional view of a brainstem-midline/intralaminar-neocortex network that implements wakefulness has recently been challenged (Fuller et al., 2011). According to Fuller et al. (2011) one of the relevant networks for state regulation starts on parabrachial glutamatergic afferents that project to the basal forebrain, which then influences neocortical state directly, and could also do so indirectly through the thalamus (Hallanger et al., 1987; Buzsaki et al., 1988; Parent et al., 1988; Steriade et al., 1988; Gritti et al., 1998). The role of the intralaminar and midline nuclei in state maintenance needs further clarification. New experimental strategies to manipulate the activity of specific pathways (Xu and Südhof, 2013) offer more selective approaches to attack this question.

Regarding cognitive behavior, the modulation of the midline and intralaminar nuclei may be important in rewarded behavior. These nuclei have high densities of dopamine D2 receptors compared to other parts of the thalamus (Rieck et al., 2004; Piggott et al., 2007); dopamine can influence the midline and intralaminar nuclei locally, but dopaminergic modulation of midline output is likely to also occur at striatal and mPFC targets. Paraventricular and dopaminergic terminals converge, in close spatial relation, onto the same cells in the nucleus accumbens, although that close relation was not found in mPFC (Pinto et al., 2003). Instead, centromedian terminals in the striatum were not found on the same postsynaptic dendrites as dopaminergic terminals (Smith et al., 1994). In mPFC, mediodorsal afferents converge on the same layer V cells contacted by dopaminergic axons, with the mediodorsal input being more distal to the soma (Kuroda et al., 1996). The anatomical data indicates that the paraventricular nucleus has the closest synaptic relation with dopaminergic terminals. The paraventricular nucleus has been suggested to participate in dopamine-mediated reward associations (Igelstrom et al., 2010; Choi et al., 2012; Martin-Fardon and Boutrel, 2012). Kelley et al. (2005) proposed that the paraventricular nucleus is an important component of the network controlling food-related, goal-directed behavior. The paraventricular would integrate energy and circadian information from the hypothalamic orexin system and relay it to the striatum to regulate dopamine levels and feeding behavior, a hypothesis that has recently received support in rats (Choi et al., 2012). In fact, the paraventricular is the thalamic nucleus with the densest orexinergic innervation (Sakurai, 2007), and the effect of these peptides on paraventricular networks deserves further investigation. The role of other nuclei in the midline and intralaminar groups (which also respond to orexins) on rewarded behavior is largely unexplored (Purvis and Duncan, 1997; Bayer et al., 2002).

**FIGURE 5:**
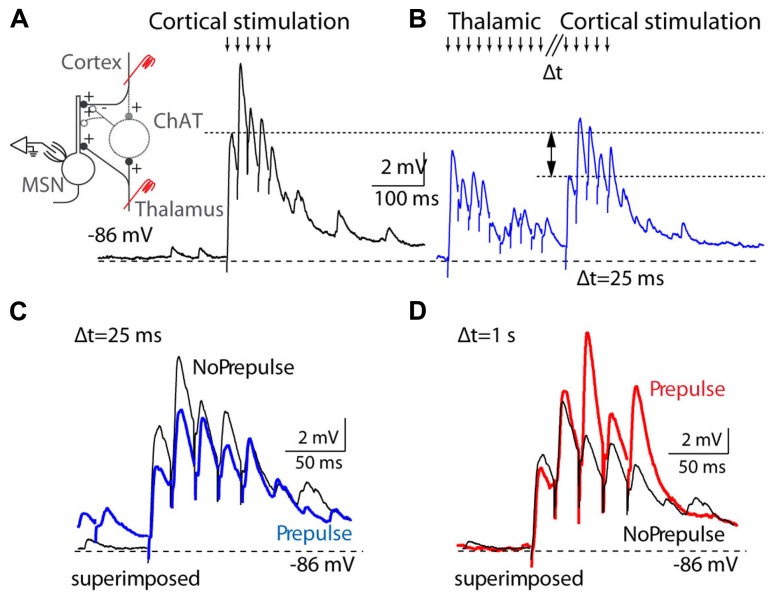
**Thalamostriatal projections gate corticostriatal inputs in mouse slices. (A)** Left, diagram of the experimental preparation: medium spiny neuron (MSN) recorded in the striatum while corticostriatal projections are activated, with or without preceding stimulation of thalamostriatal projections. Right, activation (downward arrows) of corticostriatal input evokes a train of EPSPs in a MSN cell. **(B)** Corticostriatal EPSPs are reduced when thalamostriatal stimulation precedes the corticostriatal stimulation by 25 ms. **(C)** Overlay of corticostriatal EPSPs before and after (blue) thalamostriatal activation to illustrate the changes in amplitude. **(D)** Overlay of corticostriatal EPSPs before and after (red) thalamostriatal activation, but with a long delay (1 s) between the thalamostriatal and corticostriatal activation. [Reprinted from (Ding et al., 2010), with permission from Elsevier.]

Recent evidence points to another important function of the caudal intralaminar group in behavioral flexibility and task switching in relation to sensory demands (Galvan and Smith, 2011). Lesions or inactivation of the parafascicular nucleus impair tasks that require behavioral flexibility and prevent the local increase in acetylcholine that occurs in the dorsal striatum during task shifting (Brown et al., 2010; Kato et al., 2011). Thalamostriatal afferents evoke a burst-pause firing pattern in striatal cholinergic interneurons; the cholinergic burst transiently silences corticostriatal afferents (**Figure 5**), and is followed by a facilitation of the striatopallidal output, which is thought to contribute to action suppression through the motor thalamus. This brief overriding of corticostriatal input followed by the biased activation of the striatopallidal “no-go” pathway, is thought to suppress ongoing motor output and allow for the selection of a different action (Ding et al., 2010). A complementary line of evidence indicates that intralaminar cells respond with burst discharges to a variety of stimuli, particularly to unexpected and salient stimuli, and could therefore play an important role in shifting attention and behavior under unexpected or changing conditions (Matsumoto et al., 2001), which would contribute to the deficits in cue-triggered responses observed after intralaminar lesions (Hembrook and Mair, 2011). An important experiment will be to determine if it is specifically the burst firing mode in intralaminar cells that evokes burst-pause firing in striatal cholinergic interneurons. Acetylcholine selectively hyperpolarizes intralaminar cells that project to striatum (Beatty et al., 2009) and this modulator could be critical at influencing behavioral flexibility at the thalamic level by keeping intralaminar cells in burst mode. Also interesting is that thalamostriatal projections from the caudal intralaminar nuclei are largely segregated from thalamocortical projections, whereas in the rostral intralaminar and outside of the intralaminar group, projections often collateralize to cortex and striatum (Smith et al., 2009). This suggests that modulation may occur selectively and independently for the thalamostriatal and thalamocortical caudal intralaminar networks.

Within the midline group, a few studies implicate the nucleus reuniens in behavioral flexibility and other cognitive processes (reviewed in: Cassel et al., 2013). In a water maze task, Dolleman-van der Weel et al. (2009) observed that reuniens lesions in rats did not alter memory acquisition, but made the animals more “impulsive” during retrieval. In probe trials, animals searched for the platform in the correct location, but, in contrast to controls, soon switched to searching all over the pool. Impulsive responses were also observed after reuniens lesions in rats in a multiple choice visual-response task (Prasad et al., 2013), although not in a similar task used by Hembrook and Mair (2011). Consistent with a role in behavioral flexibility, inactivation of reuniens produced deficits in behavioral paradigms that required response inhibition, like the passive avoidance task (Davoodi et al., 2011) and a task that required switching from egocentric to allocentric navigation strategies (Cholvin et al., 2013). An important confound is that inactivation of reuniens can have additional effects, such as impairment of working memory (Hallock et al., 2013) and enhancement of memory generalization (Xu and Südhof, 2013), which could produce impairments in cognitive flexibility. Outside of reuniens, there is some evidence that the mediodorsal nucleus may contribute to behavioral flexibility; inactivation of this nucleus leads to perseverative errors in a task that required rats to switch from egocentric to cue-discrimination strategies (Block et al., 2007). More research is needed to clarify the role of the midline thalamus in behavioral flexibility and to begin the exploration of thalamic modulation on this function. Evidence from mPFC (a major target of the midline nuclei) indicates an important role for dopamine in behavioral flexibility (Floresco and Magyar, 2006) and makes this modulator an inviting starting point.

## SUMMARY AND FUTURE DIRECTIONS

Most modulators have relatively similar properties within first order thalamic nuclei, but differ in either their anatomical or functional features between first and higher order. **Table 1** summarizes the key anatomical and physiological findings in first and higher order nuclei, as well as those specific to the midline and intralaminar areas. Higher order nuclei receive glutamatergic modulators from the lower sublamina of layer VI, they receive cholinergic input with a larger LDT component than first order, they have subsets of cells that are hyperpolarized by acetylcholine and serotonin, and receive denser projections from brainstem modulators (cholinergic, serotonergic, noradrenergic, and dopaminergic). Many higher order nuclei have not been extensively studied, and further research is needed to advance our understanding of the similarities and differences across nuclei, and to fully characterize their functional implications.

**Table 1:** Summary.

Modulator	Anatomy	*In vitro*	Systems	Executive thalamus
Glutamate	-Small terminals with synapses on distal dendrites	-Direct depolarization of relay cells -Pair-pulse facilitation	-Involved in gain control -Modulates receptive field properties without affecting spatial selectivity	
				
Acetylcholine	-Mostly small terminals, some similar in size to drivers and can overlap with drivers in proximal dendrites. -More projections to higher order nuclei from LDT	-Most common direct effect: depolarization -Direct hyperpolarization in subsets of cells in higher order nuclei -Can reduce inhibitory TRN input onto relay cells	-Potential contribution to attentional and state-dependent cortical activation through the thalamus	-Large cholinergic terminals in mediodorsal and parafascicular -In parafascicular, the hyperpolarization is specific to striatum-projecting cells -Mediodorsal nucleus receives cholinergic input from the basal forebrain (in addition to other sources)
Serotonin	-Atypical, “en passant” synapses, with little morphological specialization -Denser innervation in higher order nuclei	-Most common direct effect: depolarization -Hyperpolarization in subsets of cells in higher order nuclei	-Potential role in thalamic mediated modulation of cortical activation through sleep-wake cycle	-Inhibits slow AHP in midline nuclei
Noradrenaline	-Atypical, “en passant” synapses, with little morphological specialization -Denser innervation in higher order nuclei	-Depolarization in most nuclei (inhibition in motor thalamus *in vivo*) -Facilitation of thalamocortical relative to intracortical transmission in the input layers of cortex	-Increases sensory responses and synchronization between thalamic cells	-Disrupts pre-pulse inhibition in mediodorsal nucleus -Reduces slow AHP and spike frequency adaptation in paratenial cells
Dopamine	-Denser innervation in higher order nuclei -Convergence of dopaminergic terminals with thalamocortical and thalamostriatal terminals at the target site	-Depolarization in LGN -Hyperpolarization in mediodorsal		-D2 receptors highly expressed in midline and intralaminar -Increases slow AHP and promotes burst firing in mediodorsal
Histamine	-Atypical, “n passant” synapses, with little morphological specialization	-Depolarization in LGN	-Increased firing in LGN, inhibition in anterior and intralaminar	-Inhibition of baseline firing rate in intralaminar
				-Selective facilitation of thalamostriatal synapses with repetitive stimulation

One crucial aspect that has been minimally investigated in the thalamus is the integration of modulator and driver inputs in individual dendrites (although see Crandall and Cox, 2013). The view of thalamic cells as relays has been so prevalent in the literature, that complementary conceptual frameworks have been weakened or not even considered. Thinking of thalamic cells as relays is important to understand thalamic function, but other views are necessary and will stir further progress on our understanding of the thalamus. The careful organization of thalamic modulator and driver synapses along the dendrites of thalamic cells suggests an important role for thalamic cells in the integration of inputs. Corticothalamic modulators have small terminals that tend to contact relatively distal parts of the dendrites of thalamic cells. Other modulators (cholinergic, serotonergic) spread their synapses along the proximal dendrites, falling within the area of termination of drivers. The overlap of synapses in proximal dendrites may facilitate the modulation of drivers and of voltage dependent channels (such as I_T_) located in those dendritic regions (Destexhe et al., 1998). The overlap between drivers and modulators is particularly relevant in higher order nuclei; these nuclei have drivers of multiple origins (Baldauf et al., 2005; Masterson et al., 2009) that could be modulated independently, something that needs to be investigated. Furthermore, the arrangement of synapses along the dendrites of thalamic cells may be important to ensure adequate interactions between modulators. Recording from thalamic dendrites is feasible (Williams and Stuart, 2000), and recent advancements in multicolor optogenetics (Klapoetke et al., 2014) allow the specific manipulation of multiple modulator populations. Studying the interaction of multiple modulators on individual dendrites is critical to figuring out their relative contribution to cell physiology, their influence on other inputs and, ultimately, the computational functions of thalamic cells.

By far, the most broadly studied effect of thalamic modulators has been the effect on membrane potential. This focus is well justified, since changing the membrane potential switches thalamic cells between the linear “tonic” mode of response (at depolarized levels) and the non-linear “burst” mode (at hyperpolarized membrane potentials). The tonic mode is thought to be an accurate mode of information transmission, whereas the burst mode has a higher signal-to-noise ratio and can be more effective at indicating a change in incoming information. This has important implications for the gating functions of thalamic nuclei through the sleep–wake cycle, and for the generation of oscillatory rhythms in thalamocortical networks. Rhythmic burst firing due to abnormal inhibition has been suggested to interfere with thalamic function and contribute to the pathophysiology of neuropsychiatric disorders, such as schizophrenia (Llinás et al., 1999; Lisman, 2012). Some modulators (acetylcholine, serotonin) specifically inhibit cells in higher order nuclei, and dysfunction of these modulators could contribute to abnormal rhythmicity in these nuclei. The effect of modulators on membrane potential also has implications for gain control, as suggested by layer VI modulation results in the visual system. Gain control at the thalamic level could represent a form of top-down control on earlier stages of the visual pathway, like the LGN, which receive layer VI afferents from the cortical regions that they project to. Future experiments will determine if layer VI projections to the thalamus can have gain control functions in higher order nuclei. These nuclei receive reciprocal (from cortical regions they project to) and non-reciprocal (from cortical regions they receive driver input from) layer VI afferents that could contribute, respectively, to top-down and bottom-up mechanisms of gain control. Furthermore, beyond the role of modulators on excitability, there is evidence that modulators influence the response properties of relay cells through the modification of the voltage-dependence of membrane conductances (e.g., the blockade of sAHP by serotonin and noradrenaline; McCormick and Prince, 1988; Goaillard and Vincent, 2002). However, the effect of these changes in the encoding of information in the awake animal is not known.

Undoubtedly, much remains to be learned about thalamic modulation from the systems perspective. Brainstem modulators experience state dependent changes in activity, and, within states, modulators could contribute to further fine-tuning, e.g., to variations in alertness. Investigation of higher order nuclei in different behavioral states could be an effective starting point. For example, during wakefulness, higher order relay cells are more likely to fire bursts than first order relay cells (Ramcharan et al., 2005), as predicted from the *in vitro* data reviewed here. However, we do not know what changes occur in higher order nuclei throughout the sleep–wake cycle, although their strong innervation from brainstem state regulation centers suggests stronger modulations than in first order nuclei.

Within the higher order nuclei, the midline and intralaminar groups stand out as essential components of the executive networks that engage mPFC and basal ganglia. Research in these thalamic groups has lagged behind the study of the sensory thalamus. Recent evidence suggests that these nuclei have a role in modulator regulated behaviors, such as behavioral flexibility and reward directed behavior. Research in this part of the thalamus is essential to understanding executive behavior and disease. The thalamus has been a successful therapeutic target for deep brain stimulation in a number of neurological conditions, such as essential tremor (Lyons, 2011). Treatments for disorders of executive function (schizophrenia, depression) will not be able to take the thalamus into account until we understand the role of nuclei like the midline and intralaminar in executive networks.

## Conflict of Interest Statement

The author declares that the research was conducted in the absence of any commercial or financial relationships that could be construed as a potential conflict of interest.
